# Correction: Exciton dynamics from the mapping approach to surface hopping: comparison with Förster and Redfield theories

**DOI:** 10.1039/d5cp90131f

**Published:** 2025-07-17

**Authors:** Johan E. Runeson, Thomas P. Fay, David E. Manolopoulos

**Affiliations:** a Department of Chemistry, University of Oxford, Physical and Theoretical Chemistry Laboratory South Parks Road Oxford OX1 3QZ UK johan.runeson@chem.ox.ac.uk; b Department of Chemistry, University of California Berkeley California 94720 USA

## Abstract

Correction for ‘Exciton dynamics from the mapping approach to surface hopping: comparison with Förster and Redfield theories’ by Johan E. Runeson *et al.*, *Phys. Chem. Chem. Phys.*, 2024, **26**, 4929–4938, https://doi.org/10.1039/D3CP05926J.

The authors regret that Fig. 3 in the original article was incorrect as the right panel showed results from a different initial condition. The correct figure is shown here. The original figure caption and the text remain correct.

**Fig. 3:**
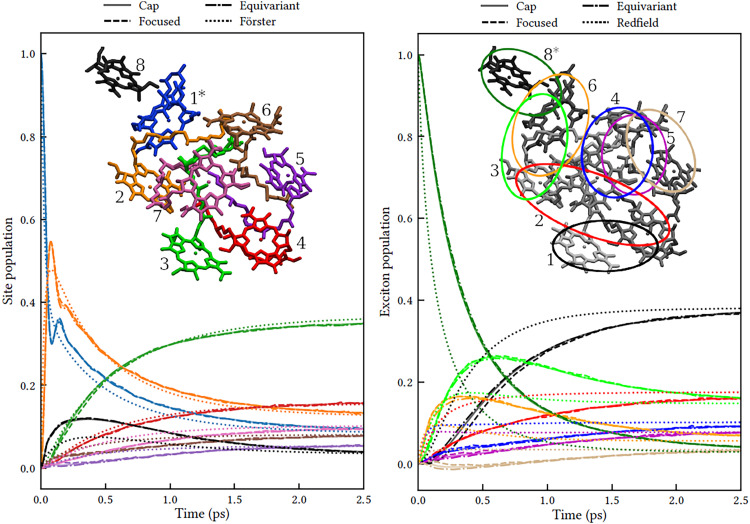
Population dynamics in FMO at 300 K comparing MASH with different initial conditions to well-established rate theories. Left: Dynamics in the site basis after an initial excitation of site 1. The three MASH initial conditions lead to identical results and agree qualitatively with Förster theory at long times. The inset shows the site labels using the same colouring as for the data curves. Right: Dynamics in the exciton basis after an initial excitation of exciton 8. The three MASH initial conditions lead to similar results and predict notably slower transfer than (secular) Redfield theory. The inset depicts qualitatively the spatial extent of the exciton states and their labels in order of increasing energy.

The Royal Society of Chemistry apologises for these errors and any consequent inconvenience to authors and readers.

